# 3D-Printed Auxetic Skin Scaffold for Decreasing Burn Wound Contractures at Joints

**DOI:** 10.3390/jfb14100516

**Published:** 2023-10-14

**Authors:** Jung-Kyu Park, Kun Woo Kim, Hyun Joo Kim, Seon Young Choi, Kuk Hui Son, Jin Woo Lee

**Affiliations:** 1 Department of Health Sciences and Technology, GAIHST, Gachon University, 155, Gaetbeol-ro, Yeonsu-gu, Incheon 21999, Republic of Korea; jungkyu_park@naver.com; 2 Department of Thoracic and Cardiovascular Surgery, Gachon University Gil Medical Center, College of Medicine, Gachon University, Namdong-daero 774 beon-gil, Namdong-gu, Incheon 21565, Republic of Korea; isee03@gilhospital.com (K.W.K.); a800313@gmail.com (H.J.K.); vet904rainbow@gmail.com (S.Y.C.); 3 Department of Molecular Medicine, College of Medicine, Gachon University, 155, Gaetbeol-ro, Yeonsu-gu, Incheon 21999, Republic of Korea

**Keywords:** auxetic, wound contracture, skin scaffold, 3D printing, electrospinning

## Abstract

For patients with severe burns that consist of contractures induced by fibrous scar tissue formation, a graft must adhere completely to the wound bed to enable wound healing and neovascularization. However, currently available grafts are insufficient for scar suppression owing to their nonuniform pressure distribution in the wound area. Therefore, considering the characteristics of human skin, which is omnidirectionally stretched via uniaxial stretching, we proposed an auxetic skin scaffold with a negative Poisson’s ratio (NPR) for tight adherence to the skin scaffold on the wound bed site. Briefly, a skin scaffold with the NPR effect was fabricated by creating a fine pattern through 3D printing. Electrospun layers were also added to improve adhesion to the wound bed. Fabricated skin scaffolds displayed NPR characteristics (−0.5 to −0.1) based on pulling simulation and experiment. Finger bending motion tests verified the decreased marginal forces (<50%) and deformation (<60%) of the NPR scaffold. In addition, the filling of human dermal fibroblasts in most areas (>95%) of the scaffold comprising rarely dead cells and their spindle-shaped morphologies revealed the high cytocompatibility of the developed scaffold. Overall, the developed skin scaffold may help reduce wound strictures in the joints of patients with burns as it exerts less pressure on the wound margin.

## 1. Introduction

Burns are one of the major injuries experienced by individuals. According to the World Health Organization, more than 11 million cases of burns occur each year worldwide [1], and approximately 180,000 fatal burn injuries occur per year [1]. The degree of a burn affects the results of skin healing; thus, the treatment of burn wounds is dependent on the severity of the burn [2,3,4]. Superficial burn wounds can be repaired with dressings; however, deep burn wounds lead to delayed wound healing and various complications [2,3,4]. Thus, deep burn wounds frequently require early excision of the necrotic tissue and wound coverage with skin grafts or skin substitutes [5]. Although the most effective treatment for deep burns is autologous skin graft, its use is limited by the availability of a skin graft source [6].

Severe burn wounds cause contracture, which forms excessive fibrous scar tissue and retracts healthy tissue. Contractures limit muscle function and reduce the range of joint motion [7]. The joints most frequently affected by burn contracture include the shoulder (23%), elbow (19.9%), ankle (13.6), and knee (13.4%) [8]. Thus, the treatment of burn wounds near joints requires further attention. Skin grafts, which consist of a thin epidermal layer and a portion of the dermis without blood supply, more frequently lead to contracture than skin flaps, which have a blood supply source [9,10,11]. Before neovascularization from the wound bed to the skin graft, oxygen or nutrients are delivered to the skin graft through diffusion from the wound bed. Thus, neovascularization of the skin graft should begin 2–3 days after surgery to ensure skin graft survival [12]. Shearing between the skin graft and wound bed should be avoided to prevent the disruption of new vessels. Therefore, the skin graft should attach very tightly to the wound bed [13]. Various methods, such as the application of fibrin glue to the wound bed [14] or tie-over bolster dressing [15], have been used to achieve skin graft stabilization. However, the tie-over bolster dressing is associated with uneven pressure distribution when a skin graft is present on an irregular wound bed [16].

Tension applied to wounds is related to wound regeneration [17]. Excessive mechanical tension leads to phenotypic transition of fibroblasts to myofibroblasts, which stimulate excessive protein synthesis and accumulation in the extracellular matrix, eventually leading to wound contracture [18]. At rest, human skin is subjected to a force of 0.4–0.98 N; however, this value increases to 0.6–2 N during the wound-healing process [19]. Mechanical stimulation is higher in keloids or hypertrophic scars, particularly at the margins of the scar [20,21]. Silicone gel sheeting has been reported to reduce keloid formation by decreasing tension at the scar margin [21].

Other skin sources, such as autografts or allograft skin, can lead to rejection [22,23]; thus, bioengineered artificial skins have been attracting attention [22,23]. Artificial skin covers the wound and acts as a bioactive dressing. Artificial skin also facilitates the wound-healing process. Thus, artificial skin should act appropriately to increase oxygen supply, maintain moisture, facilitate skin regeneration, and protect the wound bed from infections [22,23,24,25]. Bioengineered artificial skins can serve as cellular grafts in which skin cells are seeded in the biomaterial or acellular grafts that do not contain cells [22,23].

Various 3D-printing techniques have been employed to develop artificial skin. Three-dimensional printing can modulate flexibility or tailor functionality through the specific fabrication of synthetic materials [26,27]. Auxetic materials can be generated using 3D printing. As auxetic materials have a negative Poisson’s ratio (NPR), they expand in the direction perpendicular to the applied load upon stretching and shrink laterally when compressed [28]. Human skin is an auxetic material that can be omnidirectionally stretched via uniaxial stretching [29].

As auxetic materials can be stretched in all directions via unidirectional stretching, we hypothesized that NPR-patterned skin scaffolds can decrease tension, thereby increasing the margin between the artificial skin and native skin. Decreased tension near the margin of the artificial skin could cause reduced scar formation and contracture. As auxetic materials also form synclastic curvatures when bent, they exhibit good shape-fitting abilities [30,31]. Auxetic materials have been used as protectors for the elbows and knees [32]. In addition, because the skin scaffold should maintain tight adhesion to the wound bed site, we hypothesized that the auxetic skin scaffold can attach tightly to the wound bed, even on the joint area, owing to its ability to form a synclastic curvature during joint bending. To prove this hypothesis, we fabricated an NPR-patterned skin scaffold with polycaprolactone (PCL) via 3D printing and determined whether this scaffold is better at fixing onto the joint and decreasing tension than a positive Poisson’s ratio (PPR)-patterned scaffold.

## 2. Materials and Methods

### 2.1. Materials

PCL (average molecular weight (Mw), 45,000 Da; Sigma-Aldrich, St. Louis, MO, USA) was used for 3D printing, while PCL (average Mw 80,000 Da; Sigma-Aldrich, St. Louis, MO, USA) was used for electrospinning. Formic acid (FA, 99.0%; Samchun Pure Chemical Co., Ltd., Seoul, Korea) was used as a solvent. Ethanol (99.9%, Ducsan Pure Chemicals Co., Ltd., Kyunggi, Korea) was diluted to 70% and used to sterilize the scaffold.

### 2.2. Design of NPR and PPR Scaffolds

To realize the NPR effect, we used a cut-missing rib pattern and we utilized a rhombus pattern for the PPR effect. NPR/PPR skin scaffolds were created by connecting multiple patterns. A Poisson’s ratio could be adjusted by controlling the length and angle of the lines that make up the pattern (Table 1).

### 2.3. Electrospinning

To fabricate epidermal fibers, PCL powder was dissolved in FA and stirred magnetically at 22 °C overnight to prepare a 20% (*w*/*v*) PCL solution. To generate dermal fibers, PCL powder was dissolved in FA and stirred magnetically at 22 °C overnight to prepare a 35% (*w*/*v*) PCL solution. The solution was dispensed from a single-nozzle spinneret with 25 gauzes (NanoNC, Seoul, Korea) at a constant feed rate of 1.0 mL/h, temperature of 22 °C, and humidity of 12.5 ± 2.5%. Electrospinning was performed using a horizontal setup (NanoNC, Seoul, Korea). The syringe tip was positioned approximately 10 cm above a flat metallic platform. A voltage of 23–25 kV was used to charge the solutions. The fiber morphologies and diameters of the scaffolds were observed using an optical microscope (OPTIKA, Ponteranica (BG), Italy).

### 2.4. 3D Printing

The 3D model of the scaffold was visualized using Solidworks (Dassault Systemes, Ve˜lizy-Villacoublay, France). The G-code was written using MasterCAM (CNC Software, LCC, Tolland, CT, USA) and transferred to the 3D bio-printer. Printing was performed using PCL (Mw = 45,000 Da). The following optimized printing parameters were employed: nozzle diameter, 300 μm; printing temperature, 90 °C; and pressure, 250 kPa.

### 2.5. Simulation

The skin-expanding motions of the NPR and PPR patterns at the human joint zone, which were modeled using computer-aided design, were analyzed using the Ansys Workbench program (Ansys, Canonsburg, PA, USA). The structure of each pattern was assumed to be sutured to the skin, and the analysis was performed via a 1-mm expansion at the eight cardinal points of the equivalent stress.

### 2.6. Formability Test

To compare the formability of the skin scaffolds, the scaffolds were first fixed on the proximal interphalangeal (PIP) joint of the index finger, which was then bent 90°. The deformation values and forces applied by the bending of both patterns were measured. Thereafter, the degree of deformation of each scaffold was evaluated by measuring the lifting length of the scaffold before and after the bending experiment. The bending force was measured using a force-sensitive resistor (FSR, 500 g, Yh Elec, Seoul, Korea) and a microcontroller board (Microcontroller ATmega 328, Arduinom S.r.l., Monza MB, Italy).

### 2.7. Calculation of the Poisson’s Ratio

To measure the Poisson’s ratios of the two pattern types, a uniaxial tensile test was conducted. When the axial strain (ε) was stretched to 0.3, the lateral strain ($\varepsilon_{L}$) was measured. Simulations were performed based on the material properties of PCL.

### 2.8. Cell Culture

Normal human dermal fibroblasts (HDFs) were purchased from PromoCell (Heidelberg, Germany). HDFs were cultured in fibroblast growth medium-2 (PromoCell, Heidelberg, Germany) supplemented with 2% fetal calf serum, 1 ng/mL basic fibroblast growth factor, and 5 μg/mL insulin at 37 °C in a 5% CO_2_ atmosphere. At 85% confluence, the cells were seeded onto the skin grafts. HDFs passaged 7 times were used in this study.

### 2.9. Cell Viability

The PCL membrane was cut to a diameter of 1 cm and sterilized with 70% ethanol for 24 h. After soaking, the membranes were washed thrice with distilled water. For HDF attachment, 0.1 mg/mL of collagen type 1 (Sigma-Aldrich, St. Louis, MO, USA) was coated on the PCL membrane. HDFs were seeded at a density of 1 × 10^4^ cells/membrane and cultured for 7 days. The culture medium was changed every 2–3 days. The proliferation of HDFs on the skin graft was determined on days 1, 3, 5, and 7 using a Cell Counting Kit (CCK-8; Dojindo Laboratories, Tokyo, Japan), according to the manufacturer’s instructions. Briefly, fresh medium containing 10% CCK-8 reagent was added to the HDF-cultured membranes. After 2 h of incubation, the absorbance was measured at a wavelength of 450 nm using a microplate reader (SpectraMax^®^ ABS Plus; Molecular Devices, San Jose, CA, USA). The cytotoxicity of HDFs on skin grafts was assessed using a LIVE/DEAD assay kit (Invitrogen, Carlsbad, CA, USA). Briefly, the medium was removed, and the cells were washed with PBS. In accordance with the manufacturer’s protocol, 2 μM calcein AM and 4 μM ethidium homodimer-1 were diluted in PBS. The diluted reagents were added to the cell culture, which was then incubated for 1 h in the dark. Each assay was performed in triplicate. Viable (green fluorescence) and necrotic (red fluorescence) cells were visualized using a confocal microscope (LSM710; Zeiss, Jena, Germany).

### 2.10. Statistical Analysis

Data were analyzed using IBM SPSS Statistics for Windows Version 22.0 (IBM Corp., Armonk, NY, USA) and are presented as means ± standard deviations (SDs). Student’s *t*-test was performed to assess differences between groups. Statistical significance was set at *p* ≤ 0.05. The statistical significance of the data is indicated with asterisks: *, *p* < 0.05, and ***, *p* < 0.001.

## 3. Results

### 3.1. Fabrication of a Flexible Skin Scaffold

**Figure 1:**
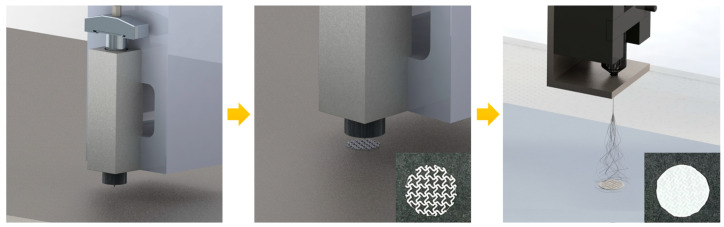
Schematic of skin scaffold fabrication.

**Figure 2:**
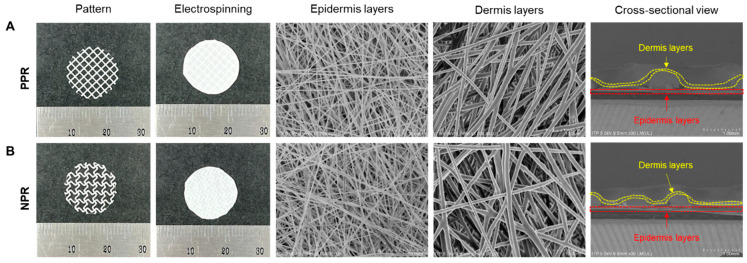
PPR- (**A**) and NPR-(**B**)-patterned skin scaffolds using 3D printing and electrospinning.

To create a highly flexible skin scaffold, 3D printing and electrospinning processes were assembled (Figure 1). A cut-missing pattern with an NPR was prepared, and a rhombus pattern with a PPR was used as a control. A circular PCL pattern with a diameter of 15 mm and a line width of 250 μm was printed to serve as a supporter of the NPR pattern and PPR pattern. Electrospinning was conducted under the same conditions for both the NPR and PPR patterns. The epidermal layer was built with 125 layers, resulting in a final thickness of 100 μm for the electrospun layer. The dermal layer was built with 150 layers, resulting in a thickness of 200 μm (Figure 2).

### 3.2. Comparison of the Poisson’s Ratios

**Figure 3:**
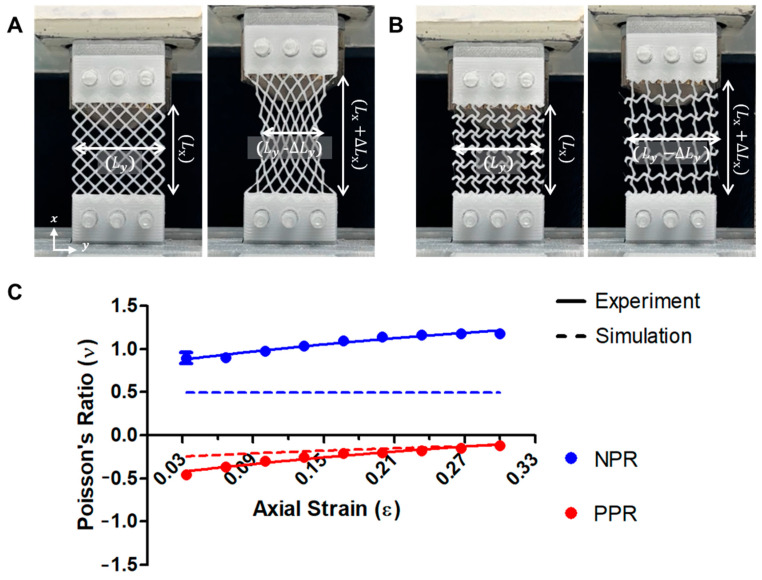
Deformation image of the PPR pattern based on the tensile stress (**A**), deformation image of the NPR pattern based on the tensile stress (**B**), and Poisson’s ratio graphs of the PPR/NPR patterns (**C**).

The Poisson’s ratio was calculated by applying tensile stress to the fabricated scaffolds and measuring their deformation. The Poisson’s ratio was defined as the ratio of lateral strain to axial strain; both the theoretical and calculated values are presented graphically. The calculated positive and negative Poisson’s ratios were similar to the theoretical values (Figure 3). When the NPR pattern was stretched by 30%, the calculated values gradually converged to −0.5 to −0.1. However, with elongation of the PPR pattern, the values displayed an increasing trend, ranging from 0.9 to 1.1. Namely, the 3D-printed skin scaffolds showed NPR and PPR values as intended in the design.

### 3.3. Simulation of the Deformation of Skin Grafts

**Figure 4:**
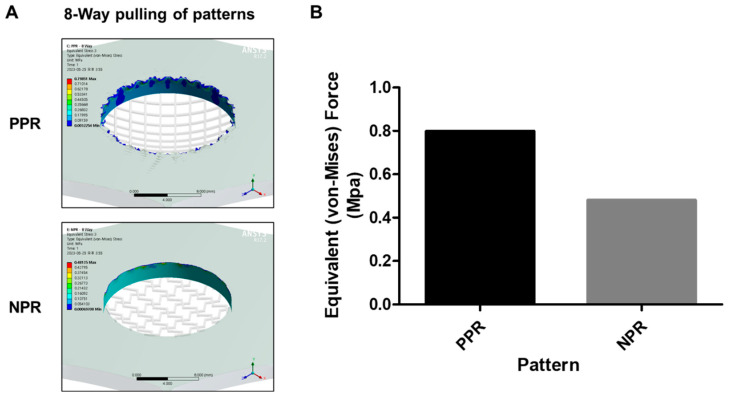
Eight-way pulling simulation results of the PPR/NPR patterns (**A**) and comparison of the loaded forces at the round edge of the PPR/NPR patterns (**B**).

When the circular pattern was assumed to be stretched in eight directions (Figure 4A,B), the force applied to the PPR pattern was 1.6-fold higher than that of the NPR pattern (Figure 4C). This result was similar to the force measurement result exerted owing to bending that was 2-fold higher value of the PPR pattern, verifying a strong correlation between the simulation and actual experiment.

### 3.4. Comparison of the Deformation and Bending Force of Skin Grafts

**Figure 5:**
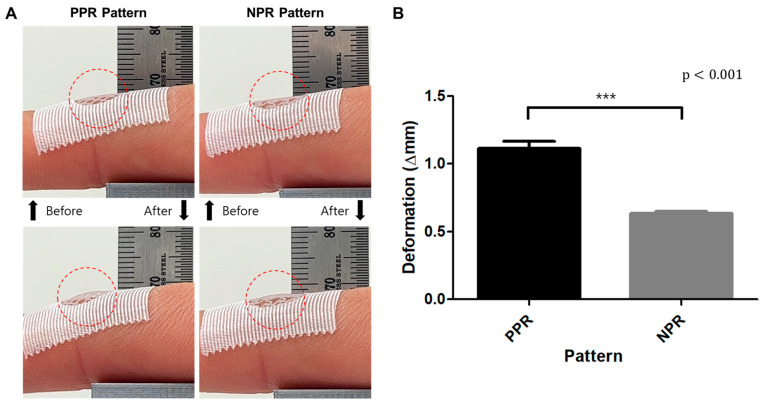
Images of grafts lifted via finger bending motions (**A**) and comparison of the deformation of the PPR/NPR-patterned grafts (**B**). (*** *p* < 0.01 compared with the PPR Pattern).

A bending test was conducted using fingers to assess the flexibility of the joint area. The performance was evaluated by measuring the lifting deformation (differences in scaffold layer positions before and after finger bending: gap between the scaffold layer and finger skin) caused by the finger-bending motion and the force exerted owing to finger bending (Figure 5A). When the deformation of the scaffolds was examined upon bending of the finger, the lifting deformation was 1.1 ± 0.13 mm for the PPR pattern, which was over 70% larger than the 0.64 ± 0.03 mm observed for the NPR pattern (Figure 5B). And the lifting deformation of NPR was statistically significantly lower than that of PPR. Namely, the NPR scaffold with a lower lifting deformation (lower gap) exhibited superior skin adherence compared with the PPR scaffold.

**Figure 6:**
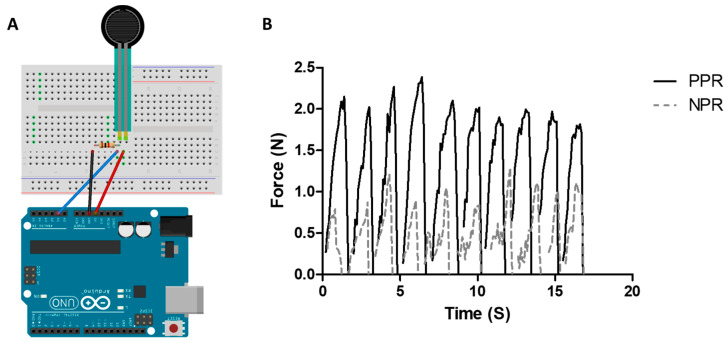
Schematic of the bending force measurement system (**A**) and measurement results of the bending forces using the NPR-/PPR-patterned patches (**B**).

When the force applied to the joint via the finger bending motion was measured, a force of 2.006 ± 0.150 N was obtained for the graft composed of the PPR pattern, whereas a force of 0.975 ± 0.087 N was obtained for the graft comprising the NPR scaffold (Figure 6). This result indicates that the PPR scaffold required more than twice the force required by the NPR scaffold to perform the same bending action. Thus, the NPR scaffold demonstrated lower contracture than the PPR scaffold as a skin graft.

### 3.5. Cytotoxicity and Proliferation of Fibroblasts on Skin Scaffold

**Figure 7:**
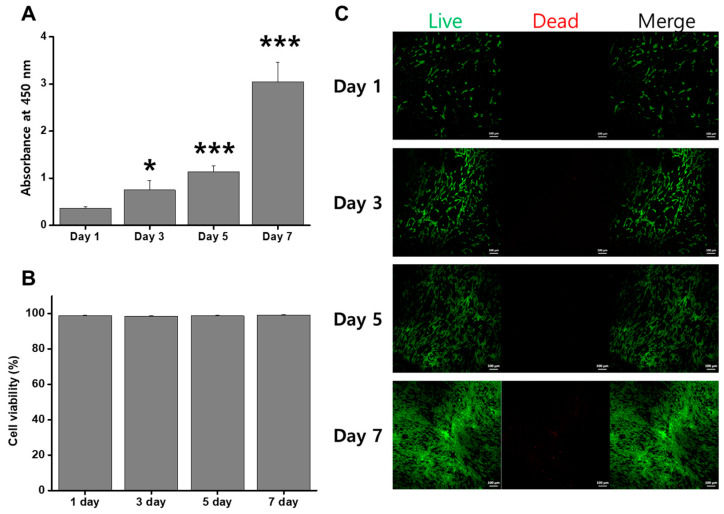
Cell proliferation and cytotoxicity of HDFs on the skin graft. CCK-8 assay (**A**) and LIVE/DEAD assay (**B**). Quantitative comparison of the live cell percentage (**C**). (* *p* < 0.05 and *** *p* < 0.01 compared with the 1-day group.).

To confirm the potential cytotoxicity induced by skin adhesion, fibroblasts were cultured on skin grafts with NPR patterns. The CCK-8 assay demonstrated that proliferation of fibroblasts was significantly increased at 3, 5, and 7 days of culture compared to that at day 1 of culture (Figure 7A).

After 7 days of culture, live cells (green fluorescence) were widely observed on the surfaces of skin grafts (>95%). Notably, dead cells (red fluorescence) were rarely observed (Figure 7B, C). In addition, the HDFs displayed a spindle-shaped morphology during all culture periods. Therefore, patterning of the skin graft did not compromise the viability of HDFs.

## 4. Discussion

The skin wound-healing process consists of four phases: hemostasis, inflammation, proliferation, and maturation (remodeling) [33]. After coagulation, inflammatory cells infiltrate the wound and promote the proliferation phase. During the proliferation phase, the wound is epithelialized, mainly by keratinocytes. Granulation tissue is generated by the proliferation of fibroblasts and formation of the extracellular matrix. During the maturation phase, type III collagen is replaced by type I collagen, and the newly generated tissue increases in strength [34]. The healing of burn wounds involves the same process. However, healing of deep burn wounds is affected by various factors. Epithelization is generally accomplished via keratinocyte migration from the wound edges [2,3,4]. Due to the shortage of keratinocytes near the wound margin, deep burn wounds frequently require a skin graft [35].

Even if epithelization is successfully completed, deep burns cause various pathological scars, such as hypertrophic scars or keloid formation [36]. Pathological scarring is induced by prolonged inflammation or abnormal wound remodeling [36]. Excessive formation of the extracellular matrix due to abnormal wound remodeling induces a hypertrophic scar, which is accompanied by pain or itching [37]. Pathological scars also cause contractures, which lead to functional limitations [37]. The frequency of hypertrophic scarring is more than 30–90% among burn injuries [37]. Based on a study that analyzed 1865 patients with burn injuries, 33% of patients were found to have at least one contracture at hospital discharge [38]. Although skin grafting is an effective treatment for burns, failure can occur due to shear-stress formation, wound infection, and inadequate skin graft size [39]. However, definitive coverage of the wound site is difficult owing to the shortage of skin grafts, especially when autologous skin grafts are used [36].

Three-dimensional (3D) printing of artificial skin is considered a solution to the shortage of autologous skin grafts. Skin constructs produced by bioprinting have been evaluated as artificial skin generation methods. However, some drawbacks are associated with the use of bioprinting. The cellular viability in a bioink is difficult to maintain [40]. Although viability can be maintained in bioinks, these bioinks are not similar to the bioelasticity of human skin [41]. Moreover, bioprinted skin has no vascular network; thus, its skin regeneration ability is limited [42].

Pores in biomaterials or scaffolds are required to improve neovascularization [43,44]. A 3D-printed skin scaffold could be a temporal wound bed cover that promotes wound healing and rebuilds mechanical stability and skin elasticity [45,46]. Various natural and synthetic materials can be used to create a 3D-printed scaffold [47]. PCL, an FDA-approved material, has good biocompatibility, a slow degradation rate, and good mechanical properties as a scaffold [48]. Owing to its slow degradation, PCL is particularly suitable for wound healing, which requires a supporting structure during skin repair [48]. PCL also has good printability, and 3D printing with PCL can be used to fabricate complex patterns for modulating mechanical properties or functionality. Previously, our group reported that a 3D-printed PCL tubular structure with an NPR pattern could increase compliance compared to the PPR pattern; thus, NPR-patterned tubular structures are more appropriate as scaffolds for artificial grafts [49]. As the NPR pattern was stretched in all directions via uniaxial stretching, similar to native skin, we hypothesized that NPR-patterned skin scaffolds can decrease contractures after burn wounds by decreasing the tensional force near the wound margin. In the experiment, similar to the simulation results, the lower tension was applied to the real margins.

Scar formation increases when the mechanical force increases, such as when the wound crosses a joint [50,51]. On the contrary, decreasing mechanical tension via tension shielding can decrease scar formation [52,53]. Thus, a skin scaffold with an NPR pattern, which is less stressful, can help reduce scar formation. The synclastic curvature created by all directional expansions/contractions of the NPR pattern has been used in various areas that require fitting to the shape [32]. Auxetic polyester fabrics show excellent fitting abilities for spherical surfaces [32]. Three-dimensionally printed thermoplastic polyurethane (TPU), which has an auxetic architecture, exhibits great deformability to fit with joints [54]. The researchers suggested that an auxetic TPU structure could be useful in pressure therapy garments [54]. Normally, pressure therapy delivers a pressure of 25 mmHg to decrease scar formation caused by garments on the wound [55,56,57]. As previously used pressure therapy garments are made of polyethylene foam with PPR, they could be displaced by joint movement and could not deliver pressure to the wound [54].

As the skin graft should be fixed to the wound bed, we hypothesized that synclastic properties can help increase the fitness of the wound bed that exists on the joint area. The skin scaffold with the NPR pattern was confirmed to have higher adhesion to the skin than the skin scaffold with the PPR pattern. Although a difference was found relative to the simulation result, which was considered ideal, the result of measuring the force applied to the pattern was also confirmed using the NPR scaffold, which was found to be half that of the PPR scaffold. In the simulation, the lower pressure on the wound margin of the NPR scaffold suggested that it facilitated wound healing and caused less contracture. The lower gap between the NPR scaffold and skin surface by the finger-bending test suggests that a skin scaffold that adheres well to the wound bed improves neovascularization during the wound healing process. Therefore, our NPR pattern is more advantageous than PPR as a scaffold when developing artificial skin, especially human skin, that will be transplanted to the joint area of patients with burns.

There are limitations to this study. First of all, we did not perform an animal study to prove our concept of which NPR pattern would be beneficial to decrease scar formation by decreasing wound tension. And, in order to be approved as a medical device, in addition to the biocompatibility obtained in this study, additional research is needed on air permeability, swelling index, biodegradability, and moisture vapor transmission rate.

Even though our study has several limitations, our results show that an NPR-patterned skin scaffold could be beneficial to reduce wound strictures in the joints of patients with burns, as it exerts less pressure on the wound margin. Clinically, wound strictures after burns, especially on joints, cause serious problems that have not yet been solved. Thus, our study is clinically meaningful by suggesting a new strategy for the treatment of burn wounds on joints via a relatively simple engineering concept. And the NPR scaffold can be used not only for artificial skin but also for reconstructing other tissues, such as muscles and blood vessels, that stretch and contract in both directions.

## 5. Conclusions

In this study, a skin scaffold with an NPR effect was fabricated by creating fine patterns through 3D printing and electrospinning. The NPR performance of this scaffold was verified through simulations and experiments. Compared to the PPR scaffold, the developed NPR skin scaffold exhibited less lifting deformation from the skin owing to the bending motion. Those results suggested that the NPR skin scaffold decreased graft dehiscence after graft transplantation, especially when the joint area was exposed to bending movement.

In the simulation and experimental results, the force applied to the scaffold margin area was lower in NPR than PPR. It suggested that the NPR skin scaffold could decrease wound margin tension, which eventually decreased scar formation and burn wound stricture. When HDFs were cultured in the NPR skin scaffold, they proliferated normally, covered the surface of the scaffold, and showed high cell viability. These results verified the biocompatibility of the developed scaffold as a graft. Our scaffold for artificial skin implantation in the joints of patients with burns will help reduce wound strictures because the NPR pattern exerts less pressure on the wound margin than the PPR pattern and less deformation due to joint movement when implanted in the joint.

## Figures and Tables

**Table 1:** Geometries of NPR/PPR patterns.

**Cut-missing rib pattern** **(NPR)**	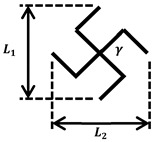	$L_{1},L_{2}=250\;\mu m$ γ=90°
**Rhombus pattern** **(PPR)**	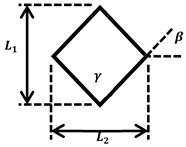	$L_{1},L_{2}=250\;\mu m$ γ=90° β=45°

## Data Availability

Data can be obtained from the corresponding author upon request.
